# Prognostic Value of Tumor Dissemination (Dmax) Derived from Basal 18F-FDG Positron Emission Tomography/Computed Tomography in Patients with Advanced Non-Small-Cell Lung Cancer

**DOI:** 10.3390/biomedicines13020477

**Published:** 2025-02-15

**Authors:** Sara Pellegrino, Rosa Fonti, Rocco Morra, Erica Di Donna, Alberto Servetto, Roberto Bianco, Silvana Del Vecchio

**Affiliations:** 1 Department of Advanced Biomedical Sciences, University of Naples “Federico II”, 80131 Naples, Italy; sara.pellegrino@unina.it (S.P.); rosa.fonti@unina.it (R.F.); ericadidonna5@gmail.com (E.D.D.); 2 Department of Clinical Medicine and Surgery, University of Naples “Federico II”, 80131 Naples, Italy; rocco.mor4@gmail.com (R.M.); alberto.servetto@unina.it (A.S.); roberto.bianco@unina.it (R.B.)

**Keywords:** tumor dissemination Dmax, 18F-FDG PET/CT, non-small-cell lung cancer, prognosis

## Abstract

**Objectives**: The aim of the present study was to test whether a parameter reflecting tumor dissemination (Dmax), derived from basal 18F-FDG PET/CT, may predict clinical outcome in patients with advanced non-small-cell lung cancer (NSCLC). **Methods**: A total of 78 patients (55 men, 23 women) with stage III and IV NSCLC who had undergone whole-body 18F-FDG PET/CT scan at diagnosis were included in this study. Imaging parameters of primary lung tumors along with total MTV (MTV_TOT_) and whole-body TLG (TLG_WB_) of all malignant lesions were determined. Moreover, the largest distance between two 18F-FDG avid lesions (Dmax) in each patient was measured. Univariate and multivariate analyses of clinical and imaging variables were performed followed by overall survival (OS) curves. **Results**: A total of 441 lesions were analyzed, including 78 primary tumors, 174 metastatic lymph nodes, and 189 distant metastases. In primary tumors, the average values of SUVmax, SUVmean, MTV, and TLG were 11.80 ± 5.73, 5.37 ± 2.09, 60.61 ± 102.57 mL, and 340.36 ± 558.40 g, respectively. The mean value of Dmax was 29.98 ± 20.98 cm, whereas the average values of MTV_TOT_ and TLG_WB_ were 155.90 ± 176.94 mL and 851.08 ± 1032.17 g, respectively. In the univariate analysis, OS was predicted by MTV_TOT_ (*p* = 0.0145), TLG_WB_ (*p* = 0.0518), Dmax (*p* = 0.0031), and stage (*p* = 0.0130), whereas in the multivariate analysis, only Dmax was retained in the model (χ^2^ = 7.3130, *p* = 0.0068). In particular, a high Dmax value indicates a worse prognosis. Moreover, the combination of Dmax with MTV_TOT_ was able to improve the prognostic stratification of patients with advanced stages of NSCLC. **Conclusions**: Dmax, by reflecting tumor dissemination throughout the body, can predict overall survival in NSCLC patients.

## 1. Introduction

Despite a significant decline in total cancer mortality, lung cancer remains the leading cause of cancer-related deaths worldwide [1]. Non-small-cell lung cancer (NSCLC) is the most prevalent histological subtype, accounting for approximately 85% of lung cancer diagnoses [2], including adenocarcinoma, squamous cell carcinoma, large-cell carcinoma, and other minor variants such as sarcomatoid cancers [3].

After pathological confirmation of the disease, the accurate staging of lung cancer represents the most relevant predictive factor of survival, guiding the subsequent therapeutic choices [4]. Current therapies of NSCLC vary from a curative intent in the early stages of the disease to different therapeutic approaches that can improve disease control and survival in patients with advanced stages while avoiding impairment of their quality of life [5,6,7]. Major advances in the biological and molecular characterization of lung cancer, as well as the elucidation of acquired resistance mechanisms, lead to the development of a large panel of new drugs [8]. Therefore, the current availability of different therapeutic agents raised the need to identify molecular markers predictive of tumor response and to achieve a more accurate prognostic stratification of NSCLC patients.

18F-Fluorodeoxy-glucose (18F-FDG) Positron Emission Tomography/Computed Tomography (PET/CT) has been successfully used for staging, treatment planning, therapy monitoring, follow-up, and prediction of clinical outcomes in NSCLC patients [9,10,11]. In addition to the visual and qualitative detection of tumor lesions, 18F-FDG PET/CT provides a variety of quantitative information that can be related to the biological features of tumors and their responsiveness to therapy. In particular, a high uptake of 18F-FDG indicated by an elevated SUVmax value is correlated with a high aggressiveness of the disease, low rates of therapy response, and a worse prognosis [12,13]. Moreover, volumetric imaging parameters such as Metabolic Tumor Volume (MTV) and Total Lesion Glycolysis (TLG) can be derived from 18F-FDG PET/CT images. When calculated on primary tumors and metastatic lesions, these quantitative parameters were reported to have a prognostic significance in NSCLC patients [10,14]. In particular, high total MTV and TLG values, by reflecting the high volumetric extension of metabolically active disease throughout the body, were found to be correlated with a worse prognosis [15].

In recent years, many studies tested the prognostic value of a number of features derived from the texture analysis of 18F-FDG PET/CT images in NSCLC patients [16]. The aim of texture analysis is to extract quantitative data that cannot be visualized but can be correlated with intrinsic biological characteristics of a tumor lesion, such as aggressiveness, heterogeneity, and resistance to therapies [17]. In particular, certain texture features such as entropy, dissimilarity, and coefficient of variation were reported to predict prognosis in NSCLC patients [16,18,19,20].

Among different quantitative PET-based imaging parameters, tumor dissemination (Dmax), defined as the largest distance between two segmented lesions on PET images, was reported to be a promising prognostic biomarker in lymphoma patients [21,22]. Therefore, the aim of the present study was to test whether this quantitative parameter, derived from 18F-FDG PET/CT, reflecting tumor dissemination (Dmax), may predict overall survival (OS) and progression-free survival (PFS) in patients with advanced non-small-cell lung cancer.

## 2. Materials and Methods

### 2.1. Patients

We studied 78 patients (55 men, 23 women; mean age ± SD: 64 ± 12 years) with newly diagnosed NSCLC who had undergone a whole-body 18F-FDG PET/CT scan at our institution from 2018 to 2022. This retrospective study was approved by the Institutional Ethics Committee (Protocol N. 352/18), and all patients signed an informed consent form for the PET/CT procedure. The eligibility criteria were histologically confirmed NSCLC, advanced disease (stages III and IV), and basal whole-body 18F-FDG PET/CT scan, performed prior to any treatment, showing at least two FDG-positive lesions suitable for segmentation (SUVmax > 2.5) in each patient. The exclusion criteria were missing imaging, histopathological, clinical, or follow-up data; previous lung or chest neoplasia; previous therapy; and the unsuitable simultaneous segmentation of at least two FDG-positive lesions.

The patients’ characteristics are reported in Table 1, showing that the majority of patients had adenocarcinoma (52%), and the most represented stage was IVB (55%).

After the basal 18F-FDG PET/CT scan, all patients were treated according to their age, stage, tumor histology, molecular pathology, PD-L1 expression, performance status, and comorbidities [6,7], as shown in Table 1.

All patients were then monitored, and the mean follow-up period was 11 ± 9 months (range 1–43 months). OS was calculated as the time between the PET/CT examination at the initial staging and the date of death. PFS was defined as the time between the PET/CT examination at the initial staging and the onset of disease progression, as assessed by the RECIST criteria or death.

### 2.2. 18F-FDG PET/CT Acquisition Protocol

Patients fasted for 8 h before undergoing the 18F-FDG PET/CT scan. Imaging acquisition was performed 60 min after the i.v. injection of 18F-FDG (370 MBq), using an Ingenuity TF scanner (Philips Healthcare, Best, the Netherlands). The following parameters were used for the CT images: 120 kV, 80 mAs, 0.8 s rotation time, and pitch of 1.5; if not previously performed, patients underwent contrast-enhanced CT. The whole-body PET scan was acquired in 3-dimensional mode using 3 min per bed position, and the number of bed positions was based on the patient’s height. An ordered subset expectation maximization algorithm was used for iterative image reconstruction. Filtered back projection of the reconstructed CT images (Gaussian filter with 8 mm full-width half maximum) was used to derive attenuation-corrected emission data to match the PET resolution. TF software (IntelliSpace Portal V5.0) was used to examine all the acquired images.

### 2.3. 18F-FDG PET/CT Image Analysis

PET/CT data in DICOM format were processed by LIFEx software (developed at CEA, Orsay, France, http://www.lifexsoft.org, accessed on 14 February 2025) [23]. 18F-FDG focal areas, covering at least 2 contiguous PET slices and not corresponding to physiological tracer uptake, were included in the segmentation procedure. In particular, the LIFEx program was used to draw a Volume of Interest (VOI) around each tumor lesion on PET images, setting an absolute threshold for SUV at 2.5 [15]. In addition, co-registered CT images were used to confirm the accuracy of lesion segmentation. Then, SUVmax, SUVmean, MTV, and TLG were obtained from all the tumor lesions. In particular, the MTV of each lesion was calculated by including all spatially connected voxels with an SUV above the absolute threshold of 2.5. TLG was obtained by multiplying the MTV by the SUVmean of each lesion. All these imaging parameters derived from primary tumors and from the targeted lesion showing the highest SUVmax value for each patient were included in the survival analysis. Moreover, total MTV (MTV_TOT_) and whole-body TLG (TLG_WB_) were calculated by the sum of MTV or TLG values obtained from each primary tumor, all the involved lymph nodes, and the distant metastatic lesions of each patient. Furthermore, the largest distance between two 18F-FDG avid lesions (Dmax) in each patient was measured. Coalescent lymph nodes were considered a single lesion. Brain metastases were not considered for lesion segmentation because of the high physiological FDG uptake in the cerebral cortex.

### 2.4. Statistical Analysis

Statistical analysis was performed using the software MedCalc for Windows, version 10.3.2.0 (MedCalc Software, Mariakerke, Belgium). Clinical and imaging variables underwent univariate and multivariate analyses for the prediction of PFS and OS, using Cox proportional hazards regression. The best discriminative thresholds of independent prognostic variables for both PFS and OS were derived by receiver operating characteristic (ROC) curve analysis. The Kaplan–Meier method and log-rank tests were used for the survival analysis.

## 3. Results

18F-FDG PET/CT images of 78 patients with advanced NSCLC showing at least two FDG-positive lesions were analyzed. Imaging parameters such as SUVmax, SUVmean, MTV, and TLG derived from the primary tumors and from the targeted lesion showing the highest SUVmax value for each patient were included in the survival analysis. In primary tumors, the average values (±SD) of SUVmax, SUVmean, MTV, and TLG were 11.80 ± 5.73, 5.37 ± 2.09, 60.61 ± 102.57 mL, and 340.36 ± 558.40 g, respectively, whereas the mean values of the same parameters in the targeted lesions were 14.74 ± 7.22, 5.71 ± 1.76, 68.89 ± 119.18 mL, and 398.95 ± 660.57 g, respectively (Table 2).

The largest distance between two 18F-FDG avid lesions (Dmax) in each patient was also obtained (Table 3). In particular, the mean Dmax value was 29.98 ± 20.98 cm, ranging from 4 to 77 cm. In 24 patients, the largest distance was observed between primary tumors and an involved lymph node, whereas in 20 patients, it was calculated between primary tumors and a distant metastatic lesion; moreover, in 34 patients, Dmax was measured between two different metastatic lesions.

To calculate MTV_TOT_ and TLG_WB_, a total of 441 lesions were analyzed, including 78 primary tumors, 174 metastatic lymph nodes, and 189 distant metastases, obtaining mean values (±SD) of 155.90 ± 176.94 mL, and 851.08 ± 1032.17 g, respectively (Table 2).

After a mean follow-up period of 11 months, 49 patients had progressive disease and died, 13 had progression and were alive, and 16 patients had stable disease. For PFS, univariate analysis was performed, including age, gender, histology, stage, and imaging parameters derived from 78 primary lung tumors and from the targeted tumor lesion, as well as Dmax, MTV_TOT_, and TLG_WB_. PFS was predicted by Dmax (*p* = 0.0061) and MTV_TOT_ (*p* = 0.0603), as reported in Table 4.

**Figure 1:**
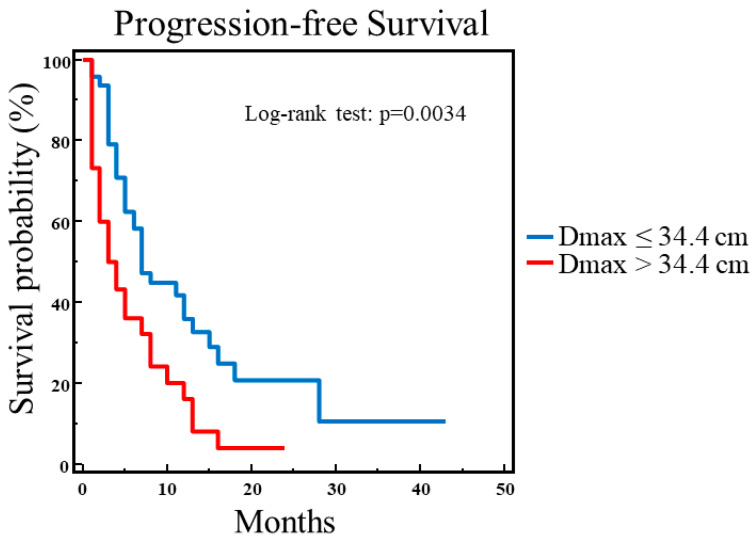
Progression-free survival analysis in 78 patients with NSCLC. A significantly prolonged PFS was found in patients with Dmax ≤ 34.4 cm as compared to those with Dmax > 34.4 cm (χ^2^ = 8.5788, *p* = 0.0034).

Then, these predictive variables along with age were included in the multivariate analysis, and only Dmax was retained in the model for the prediction of PFS (χ^2^ = 6.2730, *p* = 0.0123). The ROC curve analysis showed that the best Dmax value discriminating between patients with and without progressive disease was 34.4 cm; in particular, by Kaplan–Meier analysis, PFS was significantly prolonged in patients with Dmax ≤ 34.4 cm as compared to those with Dmax > 34.4 cm (χ^2^ = 8.5788, *p* = 0.0034) (Figure 1).

**Figure 2:**
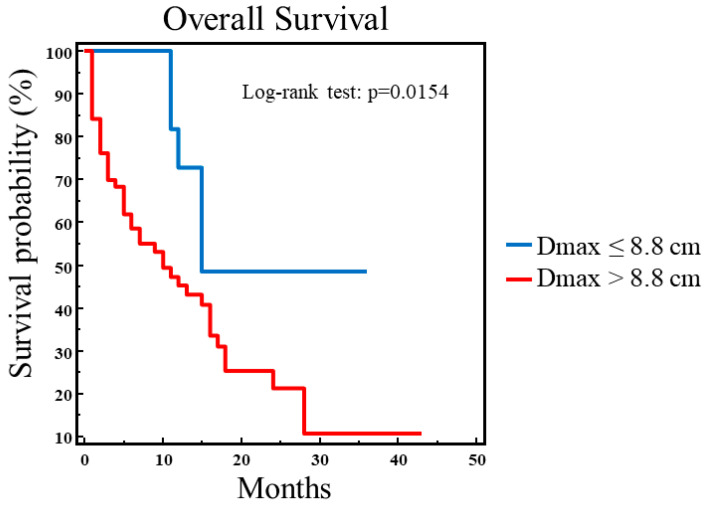
Overall survival analysis in 78 NSCLC patients. A significantly longer OS was found in patients with Dmax ≤ 8.8 cm as compared to those with Dmax > 8.8 cm (χ^2^ = 5.8673, *p* = 0.0154).

At univariate analysis for OS, we found that Dmax (*p* = 0.0031), MTV_TOT_ (*p* = 0.0145), TLG_WB_ (*p* = 0.0518), and stage (*p* = 0.0130) were significantly correlated with survival, as shown in Table 4. Then, these predictive variables along with age were included in the multivariate analysis, and only Dmax was retained in the model for the prediction of OS (χ^2^ = 7.3130, *p* = 0.0068). The ROC curve analysis showed that the best Dmax value discriminating between survivors and patients who had died was 8.8 cm. Figure 2 shows that the OS curve obtained by the Kaplan–Meier method and log-rank test was, in fact, significantly prolonged in patients with Dmax ≤ 8.8 cm as compared to patients with Dmax > 8.8 cm (χ^2^ = 5.8673, *p* = 0.0154).

**Figure 3:**
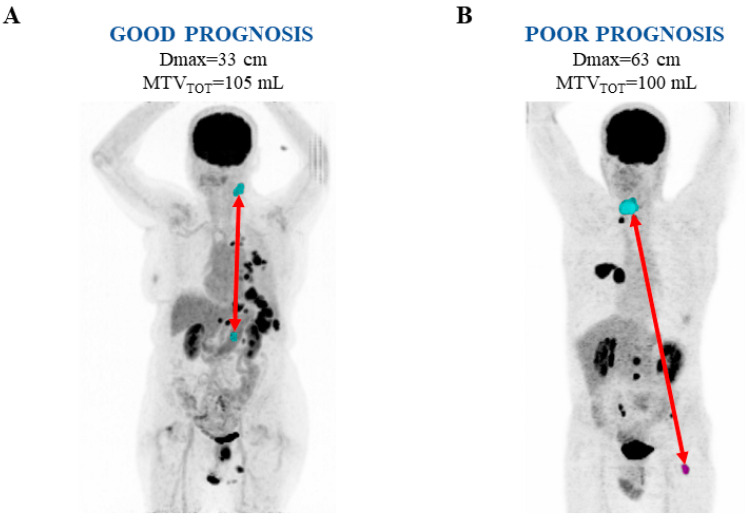
Representative images of 18F-FDG PET/CT scan in two patients with stage IV NSCLC with good (**A**) and poor (**B**) prognosis. Maximal intensity projection PET images showing the largest distance (Dmax) (red arrows) between two 18F-FDG avid segmented lesions (blue/purple areas) in each patient.

Figure 3 shows representative images of the 18F-FDG PET/CT scan in two patients with stage IV NSCLC with a good (A) and poor (B) prognosis.

**Figure 4:**
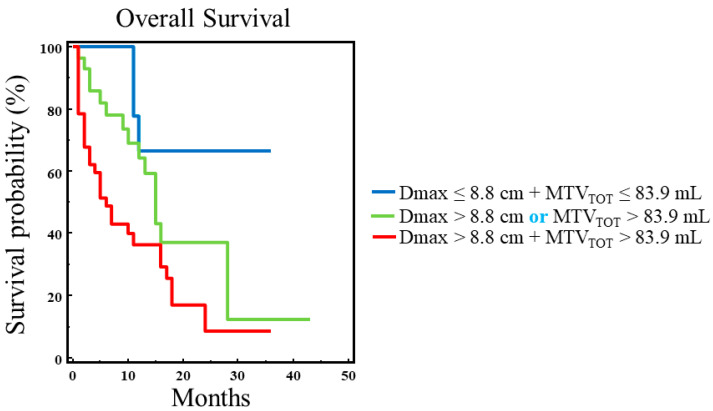
Overall survival analysis using all possible combinations of Dmax and MTV_TOT_. A statistically significant difference was found among the three survival curves (χ^2^ = 10.7451, *p* = 0.0046). In particular, the best OS was observed in patients with Dmax ≤ 8.8 cm and MTV_TOT_ ≤ 83.9 mL, while patients with both Dmax and MTV_TOT_ values above their respective thresholds had the worst prognosis.

MTV_TOT_ was not found to be an independent prognostic factor for OS but was significantly correlated with prognosis. Therefore, we combined this parameter with the Dmax value for the Kaplan–Meier analysis by using their respective thresholds. In particular, we grouped patients with only one parameter above the cut-off value (Dmax or MTV_TOT_) and then tested whether the three possible combinations could better stratify our population. As shown in Figure 4, a statistically significant difference was found among the three survival curves (χ^2^ = 10.7451, *p* = 0.0046). In particular, the best OS was observed in patients with Dmax ≤ 8.8 cm and MTV_TOT_ ≤ 83.9 mL, while patients with Dmax > 8.8 cm and MTV_TOT_ > 83.9 mL had the worst prognosis.

## 4. Discussion

Our study showed that Dmax, an imaging parameter derived from 18F-FDG PET, by reflecting tumor dissemination, is an independent predictive factor of clinical outcomes in NSCLC patients. In particular, patients with Dmax values higher than the threshold had a significantly worse overall survival as compared to patients with Dmax values lower than the cut-off. Similarly, the same parameter was able to predict progression-free survival in the same cohort of patients. Moreover, when we combined Dmax with MTV_TOT_, a parameter indicating the metabolically active tumor burden in the whole body of each patient, we found that the combination of these parameters was able to better stratify patients with advanced NSCLC. In particular, patients with the worst prognosis had both Dmax and MTV values higher than their corresponding thresholds.

Despite the high degree of interest in the extraction of quantitative variables from 18F-FDG PET/CT images by texture analysis [24,25,26,27], only a couple of studies have evaluated the ability of Dmax to predict prognosis in NSCLC patients [28,29]. Tan et al. [28] studied 101 NSCLC patients with stage IV disease receiving first-line systemic therapy and found that Dmax and whole-body MTV were independent prognostic factors for both OS and PFS. These results were partly confirmed by our findings as in our population, the multivariate analysis revealed that only Dmax was an independent prognostic factor for both OS and PFS.

Further clues on the role of Dmax were provided by Orlhac et al. [29]. In fact, they evaluated pretreatment 18F-FDG PET/CT scans of 104 patients with advanced EGFR-driven NSCLC undergoing therapy with tyrosine kinase inhibitors. The results of this study, reported in a meeting abstract, show that the addition of Dmax to MTV_TOT_ improved the prognostic stratification of patients, thus accurately predicting OS.

Recently, Dmax has been successfully applied in the prognostic stratification of lymphoma patients. Cottereau et al. [30] evaluated basal 18F-FDG PET/CT scans of 95 patients with advanced diffuse large B-cell lymphoma (DLBCL). They found that imaging parameters such as MTV_TOT_ and Dmax were significantly correlated with OS, whereas only Dmax was significantly associated with PFS. Moreover, the combination of MTV and Dmax further improved the risk stratification of DLBCL patients before therapy. Dmax has also been tested in different lymphoma subtypes, including Hodgkin lymphoma, follicular, and mantle cell lymphoma [31,32,33]. Regardless of the segmentation method used and the derived thresholds, these studies showed that Dmax was a strong predictor of both OS and PFS, suggesting its usefulness also in other tumor types.

Dmax is a simple tridimensional parameter that is operator-independent and automatically derived from image segmentation software. Moreover, the Dmax calculation is not affected by the technical acquisition parameters; therefore, it is possible to compare the values obtained with different PET scans in multicenter trials. However, some issues related to its wide clinical application remain to be addressed. For instance, it is not established whether the Dmax measurement should be normalized to the patient’s body surface area [33] or to other body characteristics. Moreover, it is debated whether Dmax may be significantly influenced by the segmentation method. Another important issue is that Dmax can be applied only to patients with at least two lesions suitable for segmentation. Other limitations of our study include its retrospective design, the limited series of patients, and the small number of patients in stage III, thus not allowing for a thorough evaluation of the prognostic role of Dmax in locoregional spread.

In conclusion, our study showed that Dmax is an independent prognostic factor for both overall and progression-free survival in patients with advanced NSCLC, and its combination with MTV_TOT_ was able to improve the prognostic stratification of our patients. Moreover, our findings indicate that Dmax should be included in the panel of quantitative parameters derived from 18F-FDG PET/CT images in patients with solid tumors since it reinforces the prognostic power of single variables.

Nevertheless, further studies are needed to standardize the method of the Dmax measurement and the selection of the appropriate threshold. Moreover, Dmax could be tested in prognostic models based on machine learning or deep learning in an effort to include this parameter in the management of different cancer patients. The use of quantitative prognostic parameters, such as Dmax, in cancer staging is key to achieving an increasingly targeted and personalized treatment in patients with both lymphoproliferative diseases and solid tumors, including NSCLC.

## 5. Conclusions

Our study showed that Dmax, representing tumor dissemination, is an independent prognostic factor for predicting overall and progression-free survival in NSCLC patients. This simple three-dimensional parameter can be easily derived and interpreted, thus providing information on the spread of a tumor throughout the whole body. Moreover, the combination of Dmax with MTV_TOT,_ that represents the whole metabolically active tumor burden, may improve the risk stratification of advanced NSCLC patients.

## Figures and Tables

**Table 1:** Clinicopathological characteristics and treatment of 78 patients with advanced NSCLC.

Characteristic	N°	%
**Patients**	78	
**Age**		
Mean ± SD	64 ± 12	
Range	38–84	
**Gender**		
Male	55	70
Female	23	30
**Histology**		
Adenocarcinoma	41	52
Squamous cell carcinoma	17	22
Large cell carcinoma	3	4
Not otherwise specified	17	22
**TNM stage**		
IIIA	2	3
IIIB	9	12
IIIC	5	6
IVA	19	24
IVB	43	55
**Treatment**		
Chemotherapy	44	56
Chemoradiotherapy	2	3
Chemotherapy/Immunotherapy	19	24
No cancer therapy	13	17

**Table 2:** PET-based imaging parameters obtained by 18F-FDG PET/CPET/CT analysis of 78 primary tumors and 78 targeted lesions showing the highest SUVmax value for each patient.

Parameters	Mean ± SD	Range
**SUVmax**		
Primary tumors	11.80 ± 5.73	3.05–38.51
Targeted lesions	14.74 ± 7.22	4.41–46.82
**SUVmean**		
Primary tumors	5.37 ± 2.09	2.72–16.37
Targeted lesions	5.71 ± 1.76	3.01–11.42
**MTV**		
Primary tumors	60.61 ± 102.57	0.26–720.96
Targeted lesions	68.89 ± 119.18	1.09–720.96
**TLG**		
Primary tumors	340.36 ± 558.40	0.69–3815.38
Targeted lesions	398.95 ± 660.57	2.72–3815.38
**MTV_TOT_**	155.90 ± 176.94	3.37–874.37
**TLG_WB_**	851.08 ± 1032.17	12.72–5428.43

SD, Standard Deviation; MTV, Metabolic Tumor Volume; TLG, Total Lesion Glycolysis; MTV_TOT_, Total Metabolic Tumor Volume; TLG_WB_, whole-body Total Lesion Glycolysis.

**Table 3:** Mean, median, and range values of Dmax derived from basal 18F-FDG PET/CT scans of 78 advanced NSCLC patients.

Dmax (Tumor Dissemination)	cm	
Mean ± SD	29.98 ± 20.98	
Median	21.6	
Range	4–77	
**Sites of lesions with the largest distance**	**Number of patients**	**%**
Primary tumor and metastatic lymph node	24	31
Primary tumor and distant metastasis	20	26
Two involved lymph nodes	7	9
Metastatic lymph node and distant metastasis	16	20
Two distant metastases	11	14

**Table 4:** Predictors of progression-free and overall survival by univariate analysis of clinical and imaging variables.

	Progression-Free Survival		Overall Survival	
Variable	χ^2^	*p*	χ^2^	*p*
**Age**	0.4640	0.4958	0.1120	0.7378
**Gender**	1.1600	0.2814	0.7130	0.3984
**Histology**	1.7000	0.1923	1.1500	0.2835
**Primary tumor SUVmax**	0.0039	0.9499	0.0035	0.9526
**Primary tumor SUVmean**	0.1450	0.7037	0.2860	0.5931
**Primary tumor MTV**	0.0495	0.8239	0.4220	0.5161
**Primary tumor TLG**	0.0709	0.7901	0.2910	0.5897
**Targeted lesion SUVmax**	0.6430	0.4226	1.2510	0.2634
**Targeted lesion SUVmean**	0.4200	0.5167	0.9140	0.3390
**Dmax**	**7.5070**	**0.0061**	**8.7230**	**0.0031**
**MTV_TOT_**	**3.5290**	**0.0603**	**5.9790**	**0.0145**
**TLG_WB_**	2.3670	0.1240	**3.7830**	**0.0518**
**Stage**	2.9810	0.0842	**6.1670**	**0.0130**

## Data Availability

The original contributions presented in this study are included in the article. Further inquiries can be directed to the corresponding author.
